# Vaccine-elicited receptor-binding site antibodies neutralize two New World hemorrhagic fever arenaviruses

**DOI:** 10.1038/s41467-018-04271-z

**Published:** 2018-05-14

**Authors:** Lars E. Clark, Selma Mahmutovic, Donald D. Raymond, Taleen Dilanyan, Takaaki Koma, John T. Manning, Sundaresh Shankar, Silvana C. Levis, Ana M. Briggiler, Delia A. Enria, Kai W. Wucherpfennig, Slobodan Paessler, Jonathan Abraham

**Affiliations:** 1 Department of Microbiology and Immunobiology, Harvard Medical School, Boston, MA 02115 USA; 2 Laboratory of Molecular Medicine, Boston Children’s Hospital, Harvard Medical School, Boston, MA 02115 USA; 3 Department of Pathology, University of Texas Medical Branch at Galveston, Galveston, TX 77555 USA; 4 Instituto Nacional de Enfermedades Virales Humanas “Dr. Julio I. Maiztegui”, Monteagudo 251 Pergamino, Buenos Aires, 2700 Argentina; 5 Department of Cancer Immunology and Virology, Dana-Farber Cancer Institute, Boston, MA 02115 USA; 6 Program in Immunology, Harvard Medical School, Boston, MA 02115 USA; 7 Department of Medicine, Division of Infectious Diseases, Brigham and Women’s Hospital, Boston, MA 02115 USA

## Abstract

While five arenaviruses cause human hemorrhagic fevers in the Western Hemisphere, only Junin virus (JUNV) has a vaccine. The GP1 subunit of their envelope glycoprotein binds transferrin receptor 1 (TfR1) using a surface that substantially varies in sequence among the viruses. As such, receptor-mimicking antibodies described to date are type-specific and lack the usual breadth associated with this mode of neutralization. Here we isolate, from the blood of a recipient of the live attenuated JUNV vaccine, two antibodies that cross-neutralize Machupo virus with varying efficiency. Structures of GP1–Fab complexes explain the basis for efficient cross-neutralization, which involves avoiding receptor mimicry and targeting a conserved epitope within the receptor-binding site (RBS). The viral RBS, despite its extensive sequence diversity, is therefore a target for cross-reactive antibodies with activity against New World arenaviruses of public health concern.

## Abstract (web summary)

The five known New World hemorrhagic fever arenaviruses use the same entry receptor, but their viral glycoproteins are markedly different. Here, the authors isolate monoclonal antibodies from a Junin virus vaccinated person that cross-neutralize Machupo virus and identify a conserved epitope in the receptor-binding domain.

## Introduction

Several groups of enveloped RNA viruses cause human hemorrhagic fevers with high case fatality rates and most lack vaccines or effective countermeasures. This underscores a critical point of vulnerability in public health. Agents that cause human viral hemorrhagic fevers include filo-, flavi-, bunya-, rhabdo-, and arenaviruses.^1, 2^ Lassa (LASV) and Lujo (LUJV) are Old World arenaviruses that cause human viral hemorrhagic fevers.^1^ Junin (JUNV), Machupo (MACV), Guanarito (GTOV), Sabiá (SBAV), and Chapare (CHPV) are arenaviruses that cause human viral hemorrhagic fevers in the New World.^1^ They are usually transmitted from rodents to humans.^3^ Clinical features of infection include hemorrhagic signs, shock, and seizures. The case fatality rate of infection ranges from 15 to 30%.^3^ New World arenaviruses also pose an emerging threat in the North America; Whitewater Arroyo virus (WWAV) was tentatively implicated in three deaths in California from 1999 to 2000.^4^

Since the 1950s, JUNV, the etiological agent of Argentine hemorrhagic fever (AHF), has caused about 30,000 cases of symptomatic disease.^3^ The live attenuated JUNV vaccine, Candid #1, induces an antibody response similar to milder forms of infection caused by wild-type (WT) JUNV and has an estimated effectiveness of 95% in humans.^5, 6^ Immunizations are administered only once in a lifetime, and the vaccine has markedly decreased the incidence of AHF since its implementation in the 1980s. AHF is unique among human viral hemorrhagic fevers because transfusion of survivor immune plasma containing high titers of neutralizing antibodies (“passive immunization”) is a highly effective treatment; it decreases the case fatality rate from 15 to 30% to <1%.^7, 8^ Neutralizing antibodies can therefore play an important role in controlling human infection.

The arenavirus surface glycoprotein complex (GPC) is the target of neutralizing antibodies. GPC comprises three functional subunits: the stable signal peptide (SSP), GP1, and GP2.^9^ GP1 binds cellular receptor(s), and GP2 contains a transmembrane domain (TM) and mediates membrane fusion.^9^ GPC assembles as a trimer of GP1/GP2/SSP heterotrimers on the virion surface.^9^ During attachment of New World arenaviruses to their cellular receptor transferrin receptor 1 (TfR1),^10–12^ a GP1 pocket accepts a tyrosine (Tyr211_TfR1_) that is conserved in all TfR1 orthologs that can support the entry of these viruses.^13^ The GP1 receptor-binding site (RBS) is the target of two murine-neutralizing antibody (GD01 and OD01) that mimic the Tyr211_TfR1_ receptor contact using an antibody complementarity-determining region (CDR).^14, 15^ The GP1 RBS and its Tyr211_TfR1_ pocket are also targeted by antibodies found in survivor immune plasma.^14^

There is substantial sequence variability in the RBS among New World arenaviruses, which probably reflects co-adaptation as they circulate in their rodent hosts.^13, 16, 17^ The sequence identity in the GP1 segment that binds to TfR1 (analogous to JUNV GP1 residues 87–235) ranges from 24 to 44% between JUNV, MACV, GTOV, CHPV, and SBAV. RBS variability is best illustrated by a disulfide-linked insert in MACV loop 10 that forms extensive contacts with TfR1 but is much shorter in the other viruses.^13, 18^ RBS variability also poses a barrier to antibody cross-neutralization; unlike well described receptor-mimicking broadly neutralizing antibodies against HIV-1 and influenza virus,^19–22^ the receptor-mimicking neutralizing antibodies GD01 and OD01 are type specific.^14, 15, 23^ The MACV loop 10 insert further restricts GP1 accessibility to cross-reactive antibodies.^23^

There is, however, anecdotal evidence that Candid #1 vaccination protects against MACV disease in humans, and although non-human primates vaccinated with an attenuated strain of JUNV (XJ clone 3) develop negligible antibody titers to MACV, they can resist heterologous virus challenge through an accelerated neutralizing antibody response against MACV.^24^ To better understand a possible role for GP1-directed cross-neutralizing antibodies in immunity conferred by Candid #1 vaccination, we used single B-cell sorting and RT-PCR to isolate GP1-reactive monoclonal antibodies from an individual who had received the vaccine 2 years prior. The two JUNV neutralizing antibodies we isolated cross-react with MACV with varying efficiency. We determined X-ray crystal structures of JUNV GP1 with the weakly cross-reactive antibody Fab and of MACV GP1 with the potently cross-neutralizing antibody Fab. These reveal that potent MACV cross-neutralization is achieved without mimicking the Tyr211_TfR1_ receptor contact and by avoiding the MACV loop 10 insert. The GP1 RBS, despite its variability, is thus a site of vulnerability for cross-reactive antibodies against these emerging human pathogens.

## Results

### GP1-directed monoclonal antibodies from a vaccine recipient

**Fig. 1:**
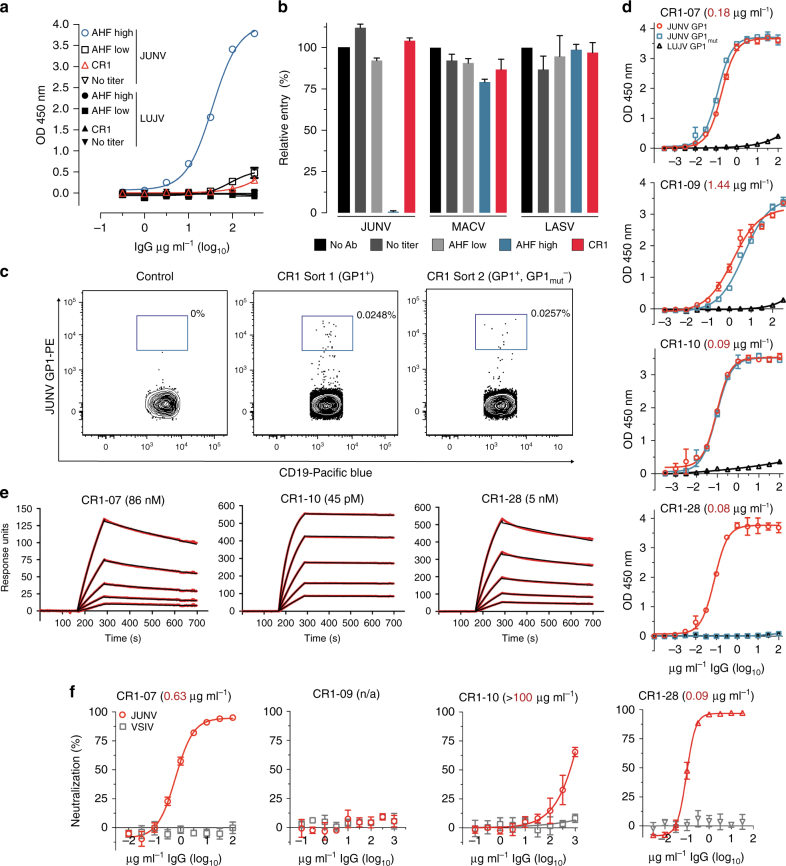
GP1-reactive monoclonal antibodies from the memory B cells of a Candid #1 recipient. **a** ELISA of polyclonal IgG purified from human plasma samples with JUNV GP1-coated plates. “AHF high”, “AHF low”, and “No titer” are AHF survivors pre-determined to have 1:10,240, 1:40, and undetectable neutralizing antibody titers (PRNT_80_) in their plasma, respectively. CR1: Candid #1 recipient. LUJV GP1 is a control. **b** HEK293T cells were infected with indicated pseudotypes after pre-incubation with polyclonal IgG (316 µg ml^−1^). Entry levels (measured by FACS for GFP) are normalized to a no antibody (“No Ab”) control. **c** Representative density plot from a FACS experiment to isolate memory B cells that bind JUNV GP1 PE tetramers (sort 1, middle panel) or JUNV GP1 PE tetramers but not JUNV GP1_mut_ PerCP tetramers (excluded in a gating step that is not shown) (sort 2, rightmost most panel). The approximate location of the sorting gate is shown as a box, and the percentage of cells that fall within the gate is indicated. The leftmost panel is for a control donor using the sort 1 strategy. CD19 is a B-cell marker. **d** ELISA of the indicated monoclonal antibodies binding to immobilized JUNV GP1 or JUNV GP1_mut_. LUJV GP1 is as a control. The calculated EC_50_ values for antibody binding to WT JUNV GP1 are indicated in parentheses. **e** Sensorgrams for binding of Fabs to immobilized JUNV GP1 as measured by surface plasmon resonance. The recorded sensorgrams (one of duplicates) are shown. Calculated *K*_D_ values are in parentheses. **f** HEK293T cells were challenged with JUNV pseudotype after pre-incubation with monoclonal antibodies. IC_50_ values are in parentheses. VSIV: vesicular stomatitis virus. For pseudotype neutralization studies, data are averaged from two independent experiments performed in duplicate. For ELISAs, the experiment was performed twice in duplicate and representative data are shown. Error bars indicate standard deviation (S.D.). For some data points, error bars are smaller than symbols

We obtained peripheral blood from an individual (CR1 for “Candid #1 recipient 1”) who had been immunized with Candid #1 2 years prior to sampling. We purified polyclonal immunoglobulins (IgG) from CR1 plasma using protein G affinity chromatography and found negligible binding to JUNV GP1 by ELISA and negligible neutralizing activity against JUNV pseudotypes, as compared to polyclonal IgG purified from the plasma of an AHF survivor known to have high-titer neutralizing activity (Fig. 1a, b). In non-human primates immunized with Candid #1, low or undetectable antibody levels prior to JUNV challenge can be boosted by JUNV infection, resulting in protection.^24^ Despite the lack of significant serological reactivity in CR1, we sought to determine if we could still identify memory B cells that encode neutralizing antibodies. Using single B-cell sorting^25, 26^ and starting with about 10 million CR1 peripheral blood mononuclear cells (PBMCs), we isolated 25 memory B cells (CD19^+^, CD27^+^, IgM^−^) that bound JUNV GP1 coupled to phycoerythrein (PE)-labeled streptavidin (SA) tetramers (Fig. 1c middle panel and Supplementary Fig. 1). We were able to subclone and express four recombinant antibodies from this first experiment (CR1-06, CR1-07, CR1-09, and CR1-10).

In a second experiment, we modified our sorting strategy to identify antibodies that, like the murine antibodies GD01 and OD01, target the GP1 Tyr211_TfR1_ pocket. For this experiment, we used PerCP-labeled SA tetramers coupled to JUNV GP1_mut_, a modified protein with Tyr211_TfR1_ pocket filling mutations (S111W, I115Y, and V117Y) that disrupt GD01 binding.^14^ Starting with about 5 million PBMCs, we isolated ten memory B cells that bound JUNV GP1 PE-tetramers but not JUNV GP1_mut_ PerCP-tetramers (included in the exclusion channel) (Fig. 1c, rightmost panel). We were able to subclone and express one monoclonal antibody (CR1-28) from this second experiment. The antibody gene usage patterns and their extent of somatic hypermutation (SHM) are listed in Supplementary Table 1.

### Antibody binding and neutralization

We tested monoclonal antibodies for binding to JUNV GP1 or GP1_mut_ by ELISA (Fig. 1d). CR1-06 did not bind JUNV GP1 (Supplementary Fig. 2a). CR1-07, CR1-09, and CR1-10 bound equally to GP1 and GP1_mut_, suggesting that their epitopes fall outside of the Tyr211_TfR1_ pocket. CR1-28 bound WT JUNV GP1 but did not bind GP1_mut_, validating the B-cell sorting strategy from which this antibody was derived. CR1-07, CR1-10, and CR1-28 Fabs bound with high affinity to immobilized JUNV GP1 as measured by surface plasmon resonance (SPR) (Fig. 1e, Supplementary Table 2). We observed weak binding of CR1-09 Fab to JUNV GP1 with a fast off rate that prevented an accurate fitting of the data to calculate binding kinetics (Supplementary Fig. 2b) In pseudotype entry assays, CR1-07 and CR1-28 neutralized JUNV with IC_50_ values of 0.63 µg ml^−1^ and 0.09 µg ml^−1^, respectively (Fig. 1f). CR1-09 and CR1-10 did not neutralize JUNV pseudotype, although CR1-10 had weak activity at high concentrations.

To determine if CR1-07 and CR1-28 bind the GP1 RBS, we next conducted competition ELISAs with GD01, a murine-neutralizing antibody that targets the GP1 RBS.^14^ CR1-07 and CR1-28 competed with GD01 for binding to GP1, suggesting that they also engage the RBS (Supplementary Fig. 3). CR1-10, however, did not compete with GD01 for binding to JUNV GP1 (Supplementary Fig. 3), suggesting that it binds an epitope that falls outside of the RBS.

### CR1-07 cross-neutralizes Machupo virus

**Fig. 2:**
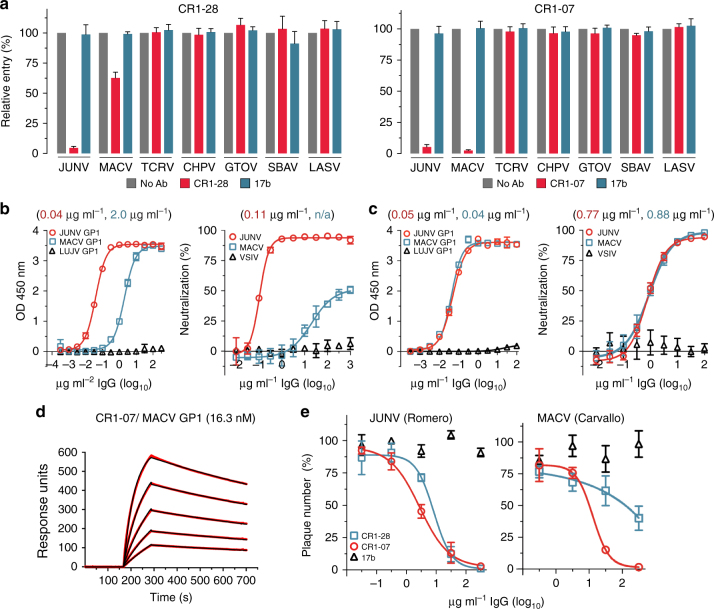
Vaccine-elicited antibodies cross-neutralize Machupo virus. **a** Pseudotypes for the indicated arenaviruses were pre-incubated with monoclonal antibodies CR1-28 (left panel) or CR1-07 (right panel) at 100 µg ml^−1^. Entry levels measured as in Fig. 1f. LASV pseudotype is included as a control. **b** Left: ELISA of CR1-28 binding to immobilized JUNV GP1 or MACV GP1. The calculated EC_50_ value is shown in parentheses. LUJV GP1 is a control. Right: HEK293T cells were challenged with JUNV or MACV pseudotypes after pre-incubation with CR1-28 at the indicated concentrations. The calculated IC_50_ value is shown in parentheses. VSIV pseudotype is included as a control. **c** Left: ELISA of CR1-07 binding to immobilized JUNV GP1 or MACV GP1. The calculated EC_50_ value is shown in parentheses. LUJV GP1 is a control. Right: HEK293T cells were challenged with JUNV or MACV pseudotypes after pre-incubation with CR1-07 at the indicated concentrations. The calculated IC_50_ value is shown in parentheses. VSIV pseudotype is included as a control. **d** Sensorgrams for binding of CR1-07 Fab binding to immobilized MACV GP1 as measured by surface plasmon resonance. The recorded sensorgrams (one of duplicates) are shown. The calculated *K*_D_ value is shown in parentheses. **e** Left: Percent plaque number (relative to a no antibody control) for infectious JUNV (Romero strain) with CR1-28 or CR1-07 at the indicated concentrations. Right: Percent plaque number (relative to a no antibody control) for infectious MACV (Carvallo strain) with CR1-28 or CR1-07 at the indicated concentrations. 17b is an HIV-1 neutralizing monoclonal antibody used as control. Error bars indicate S.D. (for some data point, error bars are smaller than symbols). For ELISAs, the experiment was performed twice in duplicate, and representative data are shown. For pseudotype neutralization studies, data are averaged from two independent experiments performed in duplicate. For studies with authentic viruses, the experiment was performed once in duplicate.

The GP1 protein of all pathogenic New World arenaviruses binds human TfR1.^10–12^ We next tested CR1-07 and CR1-28 at a concentration of 100 μg ml^−1^ for cross-reactivity against pseudotypes for the pathogenic arenaviruses MACV, GTOV, CHPV, and SBAV. Among these, JUNV and MACV are the most closely related; their receptor-binding domains (RBDs) share 44% sequence identity. We also included a pseudotype for Tacaribe virus (TCRV), a non-pathogenic virus that is closely related to MACV and JUNV (43% sequence identity with the JUNV RBD). CR1-28 and CR1-07 both had activity against MACV pseudotype but lacked activity against the other viruses we tested (Fig. 2a). CR1-28 reacted weakly with MACV GP1 in an ELISA and poorly neutralized MACV pseudotype (Fig. 2b). CR1-07, however, bound to JUNV and MACV GP1 equivalently in an ELISA and potently cross-neutralized MACV pseudotype (Fig. 2c). Its affinity for MACV GP1 as measured by SPR was 16 nM (Fig. 2d and Supplementary Table 2). In plaque reduction neutralization assays with authentic viruses, CR1-28 significantly reduced plaque formation of infectious JUNV (Romero strain) (Fig. 2e, left panel). CR1-07 reduced plaques for infectious JUNV and MACV (Fig. 2e). As has been observed with filoviruses,^27^ the potency of antibodies against infectious viruses was lower than against pseudotypes.

### Structure of a JUNV GP1-ternary Fab complex

While we could not obtain crystals of a JUNV GP1/CR1-28 Fab complex, a CR1-10 Fab/JUNV GP1/CR1-28 Fab complex crystallized in the *P*2_1_ space group (Supplementary Table 3). We determined the structure of the complex using molecular replacement with JUNV GP1 (PBD: 5EN2)^14^ as a search model and BALBES^28^ to identify Fab fragments and refined it using data extending to 4.0 Å. The complex crystallized with four copies in the asymmetric unit (ASU) allowing us to use electron density modification for phase improvement.^29^ Examples of the observed electron density at the glycoprotein-Fab interfaces are shown in Supplementary Figure 4.

**Fig. 3:**
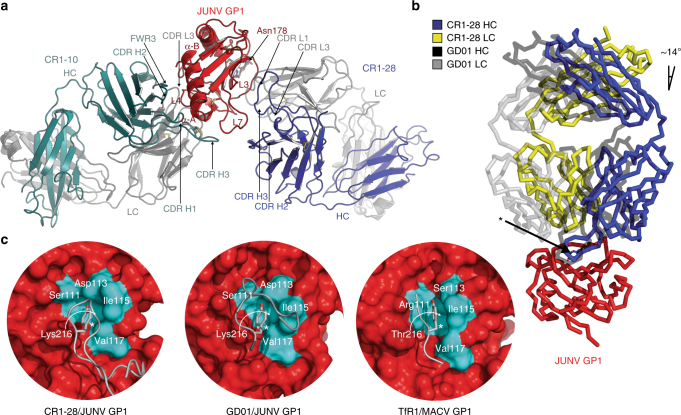
Structure of a JUNV GP1 ternary Fab complex. **a** Ribbon diagram of JUNV GP1 (red) bound to CR1-10 (heavy chain in cyan, light chain in gray) and CR1-28 (heavy chain in purple, light chain in gray) Fabs. The antibody CDRs and GP1 segments involved in interactions are labeled. L: loop. LC: light chain. HC: heavy chain. A site of N-linked glycosylation at Asn178 (for which we observed interpretable density) is shown. Disulfides are shown as yellow sticks. **b** Overlay of the JUNV GP1/GD01 Fab structure (PDB: 5EN2)^14^ with the JUNV GP1/CR1-28 Fab subcomplex. The difference in the angle of Fab approach (from the perspective of the variable domain) is indicated and is similar for all copies in the ASU. The side chain for a tyrosine that is conserved in the CDR H3 of both antibodies is shown as sticks and indicated with an arrow. **c** Surface representation of GP1 (red) with interacting segments of CR1-28 CDR H3 (leftmost panel), GD01 CDR H3 (PDB: 5EN2,^14^ middle panel), or the TfR1 βII-2 strand (PDB: 3KAS,^13^ rightmost panel) shown in cartoon representation. Residues that line the Tyr211_TfR1_ pocket are shown in cyan and labeled—the residue that lines the back of the GP1 pocket is indicated with a curved arrow. The tyrosine side chain that occupies the GP1 pocket in each instance is shown as sticks and is indicated with an asterisk

CR1-10, an antibody that binds with high affinity to GP1 (Fig. 1e) but weakly neutralizes JUNV at high concentrations (Fig. 1f), contacts the non-receptor-binding face of GP1 with four of its six CDRs along with antibody framework region 3 (Fig. 3a). Its epitope comprises GP1 loop 4, part of loop 7, and helices α-A and α-B. CR1-10 CDR H3, which contains an internal disulfide bond, forms the most GP1 contacts. To determine if CR1-10’s lack of neutralizing activity is because its epitope is buried at the trimer interface and is inaccessible on authentic JUNV GPC, we performed antibody binding studies with HEK293T cells transfected with full length JUNV GPC (Supplementary Fig. 5). This form of the glycoprotein should contain trimers of GP1:GP2 heterodimers, the TM segments, and SSP. CR1-10 bound to cells expressing JUNV GPC with an EC_50_ of 1.1 μg ml^−1^, suggesting that its epitope is available for antibody binding on trimeric GPC.

CR1-28 binds the receptor-binding face of GP1 through four of its six CDRs (Fig. 3a). CDR H3 makes most of the GP1 contacts and engages the receptor-binding groove formed by GP1 loops 3 and 7. We isolated CR1-28 with a sorting strategy that aimed to identify vaccine-elicited antibodies that, like GD01 or OD01, engage the GP1 Tyr211_TfR1_ pocket. Comparison of the GD01 and CR1-28 structures reveals that both antibodies engage GP1 with a slight shift in the angle of approach (Fig. 3b) and that CR1-28 CDR H3 also inserts a tyrosine side chain into the GP1 Tyr211_TfR1_ pocket (Fig. 3c). CR1-28 is therefore a receptor-mimicking antibody elicited by human vaccination. Unlike GD01, however, CR1-28 weakly cross-reacts with the MACV RBS.

### Structural basis for efficient MACV cross-neutralization

A complex of MACV GP1/CR1-07 Fab crystallized in the *P*4_2_2_1_2 space group, with diffraction extending to 3.9 Å (Supplementary Table 3). We were initially unable to identify a solution using molecular replacement with MACV GP1 (PBD: 2WFO)^18^ as a search model and BALBES^28^ to identify the Fab fragment. We therefore determined a high-resolution (2.0 Å) structure of the unliganded CR1-07 Fab (Supplementary Table 3 and Supplementary Fig. 6a) and used that structure, along with MACV GP1, as search models for molecular replacement. We identified four copies in the ASU allowing the use of electron density modification for phase improvement. An example of the observed electron density for the glycoprotein-Fab interface is shown in Supplementary Figure 6b.

**Fig. 4:**
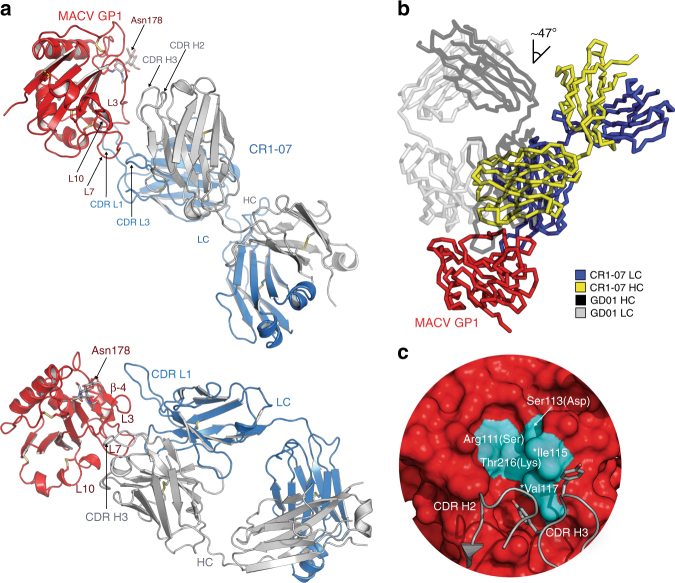
Structure of a MACV GP1 CR1-07 Fab complex. **a** Top panel: Ribbon diagram of MACV GP1 (red) bound to the CR1-07 Fab (heavy chain in gray, light chain in blue). The antibody CDRs and GP1 segments involved in interactions are labeled. Bottom panel: Side view of the complex that allows better visualization of Fab-glycoprotein interactions. A site of N-linked glycosylation at Asn178 (for which we observed interpretable density) is shown. L: loop. LC: light chain. HC: heavy chain. Disulfides are shown as yellow sticks. **b** Overlay of the JUNV GP1/GD01 Fab structure (PDB: 5EN2)^14^ with the MACV GP1/CR1-07 Fab structure. The difference in the angle of Fab approach (from the perspective of the variable domain) is indicated and is similar for all copies in the ASU. **c** Surface representation of MACV GP1 (PDB: 3KAS)^13^ with segments from the CR1-07 Fab CDRs shown in cartoon representation. Residues that line the Tyr211_TfR1_ pocket in MACV GP1 are shown in cyan and labeled. Two CDR H3 tyrosines that interact with pocket residues are shown as sticks. Asterisks indicate the two pocket residues that are conserved in JUNV and MACV GP1. For the other pocket residues, which differ between MACV and JUNV GP1, the amino acid found at the corresponding position in JUNV GP1 is shown in parentheses

**Fig. 5:**
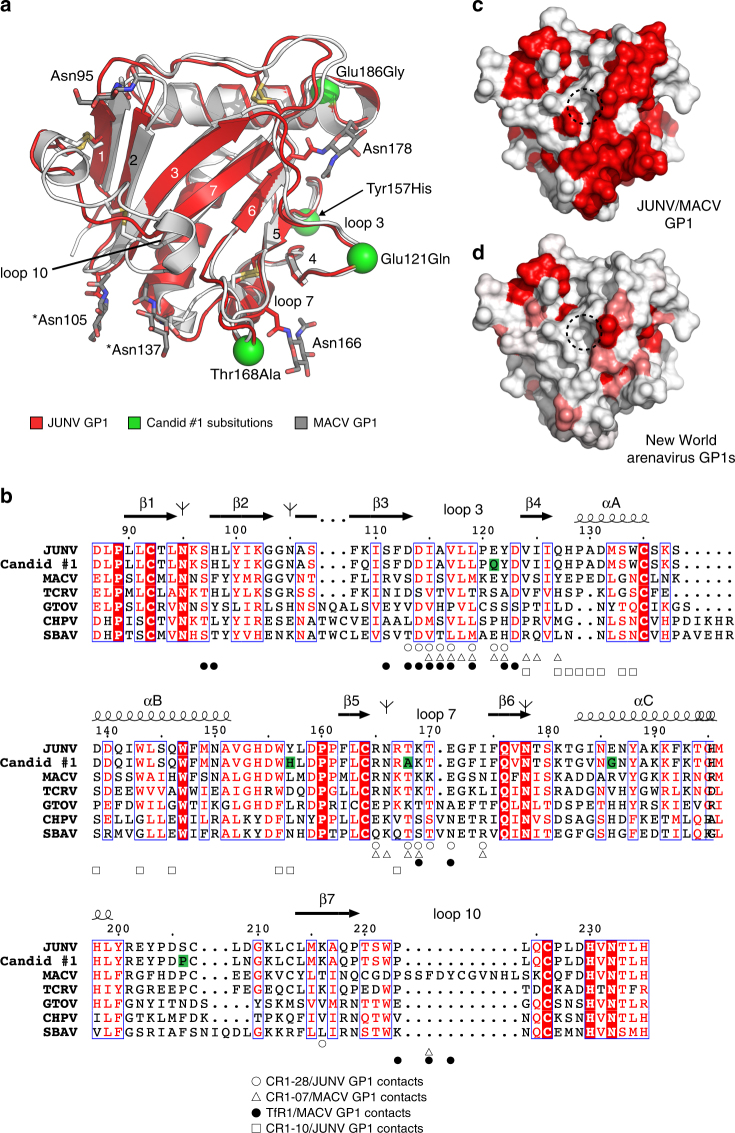
Sequence diversity in New World arenavirus GP1s. **a** Overlay of ribbon diagrams of JUNV GP1 (red, PDB: 5EN2^14^) and MACV GP1 (gray, PDB: 3KAS^13^). N-linked glycans are shown as sticks. Asterisks indicate N-linked glycans that differ in position between JUNV and MACV. Green spheres are sites of substitutions between the JUNV MC2 strain and Candid #1, the live attenuated vaccine strain. Loop 10 is a disulfide-linked insert that is unique to MACV. Disulfide bridges are shown as yellow sticks. **b** Sequence alignment of New World arenavirus GP1s. Antibody and TfR1 contacts are indicated. Substitutions in Candid #1 (with respect to JUNV MC2) are highlighted in green. Tree diagrams indicate predicted sites of N-linked glycosylation for JUNV GP1. The figure was generated using ESPrit3^64^ and modified. GenBank accession numbers used for sequences used for the alignment are JUNV (MC2, D10072), JUNV (Candid #1, ACY70854.1), MACV (Carvallo, NC_005078), GTOV (INH-95551, NC_005077), CHPV (810419, YP_001816782.1), and SBAV (SPH114202, NC_006317). The sequence of TCRV shown in the alignment is that of the pseudotype used in this study and other studies,^14, 30^ which has a deletion spanning amino acid residues 121–132 and three substitutions (I134A, G418S and E458R) compared to GenBank NC_004293. **c** Surface representation of JUNV GP1 (PDB: 5EN2)^14^ showing conservation based on the alignment with MACV GP1 shown in **b** generated with ESPrit3.^64^ Dashed circle indicates the location of the Tyr211_TfR1_ pocket. **d** Surface representation of JUNV GP1 (PDB: 5EN2)^14^ showing sequence conservation with MACV, GTOV, CHPV, SBAV as shown in **b**. Using the algorithm available in ESPrit3,^64^ residues with a similarity global score lower than 0.7 are in white, and residues with a similarity global score in the range 0.7–1.0 are color-ramped in shades of red (with dark red showing the greatest conservation)

The binding mode of the CR1-07 Fab is different from that of GD01 or CR1-28; CR1-07 CDR L1, which is 14 residues long, protrudes from the CDR surface and dominates the interaction with GP1 (Fig. 4a). CDR L1 and CDR H3 clasp the C-terminal segment of GP1 loop 3, and CDR L1 forms additional contacts with the GP1 β-4 strand. Comparison with the GD01 structure shows that the angle of approach is different and that the tip of CR1-07 CDR H3 shifts away from the GP1 Tyr211_TfR1_ pocket (Fig. 4b). Instead of engaging the cavity, two tyrosines in CR1-07 CDR H3 interact with Ile115 and Val117 on the rim of the pocket; these are the only two pocket residues conserved in MACV and JUNV GP1; MACV GP1 residues Arg111, Ser113, and Thr216 are respectively replaced by a serine, aspartate, and lysine in JUNV GP1 (Figs. 4c and 5). The other GP1 residues CR1-07 contacts are conserved between JUNV and MACV GP1, but not among the other viruses (Fig. 5 and Supplementary Fig. 7). CR1-07 binds with high affinity to JUNV GP1_mut_, a construct in which Ile115 and Val117 are replaced by tyrosines (Fig. 1d). This suggests that CR1-07 would nonetheless tolerate substitutions at these positions and that most of its binding energy is contributed by conserved contacts elsewhere in the RBS.

**Fig. 6:**
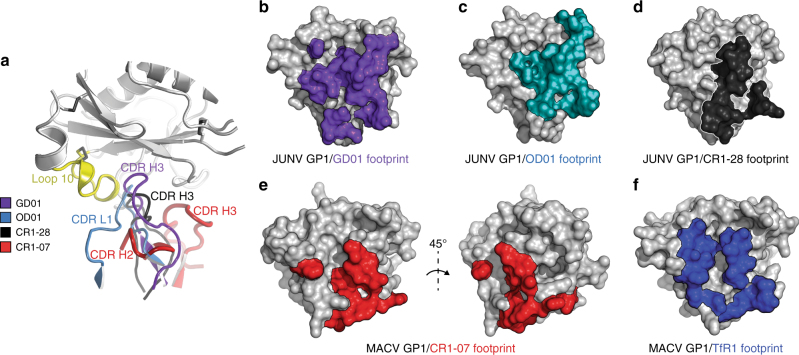
Mechanism for efficient MACV cross-neutralization. **a** Ribbon diagram of MACV GP1 (gray) with loop 10 shown in yellow, and CR1-07 CDR H2 and CDR H3 (as determined in the MACV GP1/CR1-07 Fab complex) in red. To allow for comparison of antibody CDR segments, the structures of the JUNV GP1/GD01 Fab complex (PDB: 5EN2),^14^ of the JUNV GP1/OD01 complex (PDB: 5NUZ),^15^ and of the JUNV GP1/CR1-28 Fab subcomplex were overlaid onto MACV GP1 by aligning the GP1s. JUNV GP1 used for alignment of the complexes is omitted for clarity. **b** Surface representation of JUNV GP1 (PDB: 5EN2,^14^ gray) with GD01-interacting residues shown in purple. **c** Surface representation of JUNV GP1 (gray) with OD01-interacting residues shown in light blue. **d** Surface representation of JUNV GP1 (gray) with CR1-28-interacting residues shown in black. **e** Surface representation of MACV GP1 (gray) with CR1-07-interacting residues shown in red. **f** Surface representation of MACV GP1 (PDB: 3KAS,^13^ gray) with TfR1-interacting residues shown in dark blue

Because of the manner through which the antibody loops of GD01, CR1-28, and OD01 engage the GP1 Tyr211_TfR1_ pocket, their side chains would clash with the unique MACV loop 10 insert (Fig. 6a). Among receptor-mimicking antibodies, because of the slight tilt in the angle of approach of the CR1-28 Fab, the clashes between its CDR loop and MACV loop 10 would be the least severe, perhaps explaining CR1-28’s ability to weakly cross-react with MACV. On the other hand, the drastically different angle of approach of the CR1-07 Fab allows its CDRs to avoid any clashes with MACV loop 10. Efficient cross-neutralization is thus achieved by avoidance of the MACV loop 10 insert, a unique segment that best exemplifies the extensive sequence variability seen within the RBS of New World arenaviruses (Fig. 5).

## Discussion

All described antibodies that neutralize New World arenaviruses engage the GP1 RBS (Fig. 6b–f). AHF survivor plasma also contains RBS-targeting antibodies and a large and accessible RBS could in part explain why transfusion of survivor immune plasma is highly effective against JUNV.^14^ The GP1 RBS, however, varies significantly among the viruses, likely reflecting their long-term co-adaptation with their respective rodent natural hosts (Fig. 5).^13, 16, 17^ We describe here two RBS-directed antibodies that cross-react with two New World arenaviruses and were elicited by vaccination against JUNV. These antibodies bind the MACV GP1 RBS with varying efficiency, which is reminiscent of the varying affinity with which various host-species TfR1 orthologs interact with different New World arenavirus GP1s.^30–32^ While JUNV and MACV, the two most closely related pathogenic New World arenaviruses, share patches of conservation in their RBS, this site is even more poorly conserved among the other pathogenic New World arenaviruses (Fig. 5c, d). This diversity poses a significant barrier to eliciting broadly neutralizing antibodies that target the RBS.

The GP1 proteins of New World and Old World arenaviruses have a similar structural fold.^33, 34^ The recently determined structure of the LASV GPC ectodomain in the prefusion conformation (a trimer of Fab-bound GP1:GP2 heterodimers, without the GPC TM or SSP)^35^ allows us to predict the geometry of receptor and antibody binding. With respect to the three-fold axis of the trimer, the angles of approach of the receptor-mimicking antibodies (GD01, OD01, and CR1-28) would be similar to that of TfR1 (Supplementary Fig. 8). CR1-07 would approach GPC with a steeper angle, with its CDR L1 forming contacts near the trimer apex (Supplementary Fig. 8c).

CR1-10 is extensively somatically hypermutated (11% for V_H_ and 3.2% for V_L_; Supplementary Table 1) and was elicited by immunization with a live vaccine (Candid#1) that should express trimeric JUNV GPC. When modeled onto the LASV trimer structure, the CR1-10 epitope would comprise part of the GP1 trimerization surface (Supplementary Fig. 8d). Without a conformational change, the CR1-10 epitope might not be available for antibody binding (or as an epitope for B-cell affinity maturation). CR1-10’s ability to bind cell surface expressed JUNV GPC (Supplementary Fig. 5), however, suggests that its epitope can at least be transiently exposed. A partially “open” trimer as a conformational state is therefore probably accessible on JUNV GPC. It may be even more available on Candid #1 GPC, as mutations it contains are thought to destabilize the metastable conformation of the glycoprotein.^36^ Favoring a model in which the arenavirus GPC is dynamic, LASV GPC is known to sample different conformations (dependent on low pH)^34, 37^ and the LASV GPC ectodomain (without the TM segments or SSP) could only be crystallized as a trimer when bound to an antibody Fab that stabilizes this oligomeric state.^33^

CR1-10 IgG binds to cells transfected with JUNV GPC with an EC_50_ of 1.9 μg ml^−1^ (Supplementary Fig. 5), which when compared to the ELISA data we obtained with soluble GP1 (0.09 µg ml^−1^; Fig.1d), suggests that CR1-10 binds full length JUNV GPC less well than JUNV GP1. Although the assays are not directly comparable, the discordance in apparent affinity is consistent with a model in which CR1-10’s lack of neutralizing activity may be due to poor epitope accessibility. Given its predicted geometry of GPC binding and its inability to compete with GD01 (a receptor-mimicking antibody) for GP1 binding (Supplementary Fig. 3), another explanation could be that CR1-10 fails to block receptor engagement (Supplementary Fig. 8a and d). While binding such a site on full length GPC leads to poor neutralization, CR1-10 binding could at least potentially allow it to participate in non-neutralizing antibody effector functions, including antibody dependent cellular cytotoxicity and complement fixation.^38^

Strategies targeting the viral receptor-binding domain to prevent receptor attachment may be a useful approach in passive immunity against other hemorrhagic fever viruses. The filovirus Ebola virus (EBOV) is probably a more difficult neutralizing antibody target than is JUNV; its receptor-binding subunit (also called GP1) contains a large and heavily glycosylated mucin-like domain (MLD) that protects its RBS until the MLD is cleaved by cathepsins in acidified endosomes during entry.^39–41^ Unlike with JUNV infection, transfusion of survivor plasma is not effective against EBOV in humans, probably in part because the transfused plasma contain low titers of neutralizing antibodies.^42, 43^ ZMapp, an antibody cocktail that comprises three humanized murine antibodies targeting epitopes that fall outside of the RBS, protects 100% of non-human primates 5 days out from viral challenge.^44, 45^ A single-human monoclonal antibody suspected to neutralize EBOV by blocking receptor attachment,^46^ when administered at a similar time point, protects non-human primates from lethal EBOV challenge.^47^ This finding suggests that antibodies that block receptor binding might be more potent in vivo against EBOV.

A limitation of our study is that the structures we report are of low resolution (4.0 Å and 3.9 Å for the JUNV and MACV complexes, respectively), so our ability to specify the details of intermolecular interactions (e.g., polar side-chain contacts) may be limited. For the JUNV complex in particular, crystals grew as thin, poorly diffracting plates, and intensities for higher resolution reflections were weak (Supplementary Table 4). For structure determination with both complexes, however, the presence of four copies in the ASU allowed us to use electron density modification for phase improvement. For the MACV complex, our model is further supported by the high-resolution structure of the unliganded CR1-07 Fab we used for molecular replacement.

Polyclonal IgG from the donor we studied (CR1) lacked detectable reactivity 2 years after immunization (Fig. 1a, b). CR1 could have either failed to seroconvert after vaccination or have had titers that waned below the limit of detection by the time of sampling. In a study examining 53 Candid #1 vaccines, antibodies reached peak or near peak values by 1 month after vaccination and remained relatively constant throughout the first nine months before waning; seroconversion was undetectable in 12 of the recipients.^48^ A lack of pre-existing antibody titers in CR1, however, does not necessarily imply lack of protection. In non-human primates immunized with Candid #1, low or undetectable antibody levels prior to a challenge are boosted following JUNV infection, resulting in protection.^24^ Such a scenario would be similar to what is observed with the hepatitis B virus (HBV) vaccine; immunized individuals can lose antibody titers within a few years, but HBV-specific memory B cells persist in the absence of serum antibodies and can provide rapid protective antibody responses upon exposure to the virus.^49^

There is anecdotal evidence that Candid #1 vaccination protects against MACV disease in humans. Our results allow us to speculate that cross-reactive amnestic memory B-cell responses (targeting the GP1 RBS) could play a role in cross-protection. This would explain how non-human primates vaccinated with attenuated JUNV, despite negligible antibody titers to MACV, can mount an accelerated and protective neutralizing antibody response against MACV.^24^ The significant sequence divergence between the GP1 proteins of JUNV and MACV could favor the recall of memory B cells that bind conserved epitopes within the exposed RBS. Such a model has been proposed to explain a recall response in humans that favors broadly cross-reactive memory B cells following pandemic H1N1 influenza virus vaccination in humans.^50^

We describe first in class antibodies that can cross-react with two New World arenaviruses. Given the reliable efficacy of neutralizing antibody-containing plasma in treating acute JUNV infection in humans,^7, 8^ our findings have important implications to the development of broadly active monoclonal antibody-based therapeutics and vaccines against JUNV, MACV, and other New World arenaviruses of public health concern.

## Methods

### Cells and plasmids

We maintained HEK293T (human embryonic kidney, ATCC CRL-1268) cells in Dulbecco’s modified Eagle’s medium (DMEM) supplemented with 10% (v/v) fetal bovine serum (FBS) for adherent cells and in Hybridoma-SFM expression medium (Life Technologies) for cells grown in suspension. We maintained HEK293S GnTI^−/−^ cells (ATCC CRL-3022) in serum-free medium (Freestyle^TM^ 293 Expression Medium, Life Technologies), and Expi293F^TM^ cells (Thermo Fisher Scientific) in serum-free medium (Expi293™ Expression Medium, Thermo Fisher Scientific). We used Vero cells (*Cercopithecus aethiops* kidney, ATCC CCL-81) for studies with infectious viruses. The absence of mycoplasma contamination was confirmed using a Universal Mycoplasma Detection Kit (ATCC). GPC-expressor plasmids for JUNV, MACV, GTOV, SBAV, CHPV, TCRV, LASV, LUJV, and VSIV have been previously described.^10, 14, 30^ We expressed secreted glycoproteins in the pHLsec vector^51^ and expressed human monoclonal antibodies (containing a 6-histidine tag at the C terminus of the heavy chain) and Fab fragments using a pVRC8400 vector expression system as previously described.^52^

### Single B-cell sorting and antibody cloning

This study was approved by the Boston Children’s Hospital Institutional Review Board (IRB-P00011143), as was the use of healthy donor control PBMCs (IRB-P00009521). We obtained written informed consent from a healthy adult male participant (CR1) who had been immunized with Candid #1 2 years before blood donation. We isolated donor PBMCs using Ficoll-Paque centrifugation and stained and sorted cells as previously described using a BD fluorescence-activated cell sorter Aria II,^25^ with the exception that we omitted the B-cell enrichment step and used JUNV GP1 Streptavidin-PE (Caltag) and JUNV GP1_mut_ Streptavidin-PerCP (BD Biosciences) as antigens in the experiment. Briefly, cells were washed, counted, and resuspended in phosphate-buffered saline (PBS) containing 2% (v/v) FBS. We adjusted cells to a density of 5 × 10^6^ cells in 100 µl and incubated them with antigen tetramers at a concentration of 0.10 μg ml^−1^ on ice for 30 min with intermittent gentle vortexing. We added antibodies CD19-Pacific-Blue (AbD Serotec/BioRad catalog number MCA1940GA) at a concentration of 40 μg ml^−1^, CD27-fluorescein (BD Biosciences catalog number 340424) at a concentration of 30 ng ml^−1^, 20 μl of IgM-allophycocyanin (BD Biosciences catalog number 551062) per 1 × 10^6^ cells, CD3-PerCP (BD Biosciences catalog number 340663) at a concentration of 480 ng ml^−1^, CD14-PerCP (BD Biosciences catalog number 345786) at a concentration of 0.8 μg ml^−1^, 7AAD (BD Biosciences catalog number 559925) at a concentration of 0.8 μg ml^−1^, and CD16 PerCP (Biolegend catalog number 302029) at a concentration of 8 μg ml^−1^ and kept the cells on ice for an additional 30 min. We then washed cells three times in PBS containing 2% (v/v) FBS and passed the cell suspension through a cell strainer prior to sorting. The gating scheme is shown in Supplementary Figure 1. To obtain the IgH and Igκ genes from sorted memory B cells, we performed single-cell cDNA synthesis using SuperScriptIII reverse transcriptase (Invitrogen) followed by nested PCR amplification of the IgH and Igκ variable regions as previously described.^25^ Our low recovery rates for paired re-arranged heavy and light chain segments from single memory B cells (4/25 in experiment 1; 1/10 in experiment 2) could possibly be explained by technical limitations, the possibility that some cells encoded IgA instead of IgG (we only selected for IgM^−^ cells), or false positive events in the flow cytometry. After sequencing PCR products, we used IgBLAST^53^ (http://www.ncbi.nlm.nih.gov/igblast) and IMGT®^54^ (http://www.imgt.org) to analyze *IgG* gene usage and the extent of V_H_/Vκ somatic hypermutation. We then cloned the PCR amplified variable segments into the pVRC8400 vector for expression of IgG and Fab constructs as previously described.^52^

### Human polyclonal immunoglobulin purification

We obtained de-identified previously collected plasma samples from survivors of Argentine hemorrhagic fever from the immune plasma bank at the Instituto Nacional de Enfermedades Virales Humanas (INEVH), based in Pergamino, Argentina. Provision of samples was approved by the INEVH Ethics Committee and the Harvard University Faculty of Medicine Committee on Human Studies (identified as not involving human subjects under 45CFR46.102(f)). We purified polyclonal IgG from de-identified Argentine hemorrhagic fever survivor plasma samples and from CR1 plasma with Protein G Ultralink Resin (Thermo Fisher Scientific) as previously described.^14^

### Protein expression and purification

For ELISAs and SPR-binding assays, we subcloned into the pHLsec vector^51^ constructs for JUNV GP1 (MC2 strain, GenBank accession number D10072 residues 87–235), JUNV GP1_mut_ (MC2 strain, D10072, residues 87–235 with mutations S111W, I115Y, and V117Y), MACV GP1 (Carvallo strain, NC_005078, residues 87–250), or LUJV GP1 (NC_012776.1, residues 59–217) containing an N-terminal His_6_-tag, followed by a Tobacco Etch Virus (TEV) protease site, a BirA ligase site, and a 7 residue linker. We transfected HEK293T cells grown in suspension with linear polyethylenimine (PEI), purified proteins using nickel affinity purification, and removed the His_6_-tag with TEV digestion followed by reverse nickel affinity purification and size-exclusion chromatography on a Superdex 200 (S200) column (GE Healthcare Life sciences). We biotinylated proteins with BirA ligase as previously described.^14^ We expressed recombinant human monoclonal antibodies and Fab fragments (in the pVRC8400 vector expression system) as previously described.^52^ We produced GD01 IgG and 17b IgG from their respective hybridomas using Protein G Ultralink^®^ Resin (Thermo Fisher scientific) and by following the manufacturer’s protocol. The final step included size exclusion on a S200 column, in which recombinant glycoproteins, antibodies, and Fabs eluted as single peaks at the expected retention volume. Recombinant proteins were >98% in purity as determined by SDS-page gel electrophoresis.

For crystallography, we subcloned JUNV GP1 (MC2 strain, GenBank accession number D10072, residues 87–229) and MACV GP1 (Carvallo strain, NC_005078, residues 87–240) along with an N-terminal His_6_-tag, a TEV protease site, and a short linker (amino acids SGSG), into the pHLsec vector.^51^ We produced proteins by PEI transfection of GnTI^−/−^ HEK293S cells grown in suspension, purified proteins using nickel affinity purification, and removed the His_6_-tag with TEV digestion followed by reverse nickel affinity purification and size-exclusion chromatography on a S200 column. We produced monoclonal antibodies by PEI transfection of Expi293F^TM^ cells (Thermo Fisher Scientific) grown in suspension. We affinity purified monoclonal antibodies using Pierce™ Protein A Agarose (Thermo Fisher Scientific) or MabSelect SuRe resin (GE Health Care Life Sciences) using the protocol specified by the manufacturer, followed by a size-exclusion step on a S200 column.

### Pseudotype transduction and antibody neutralization

We packaged virus pseudotypes in HEK293T cells by transfecting plasmids expressing the murine leukemia virus *gag/pol*, arenaviral GP, and the pQXIX transduction vector (BD Biosciences) expressing enhanced green fluorescent protein (eGFP) in a 1:1:1 ratio, as previously described.^10^ We harvested culture supernatant containing virus at 24 h and 48 h post transfection, filtered these supernatants through a 0.45 μm membrane, pooled and stored them at −80 °C. For antibody neutralization experiments, we incubated pseudotypes with either polyclonal or monoclonal IgG in culture media containing 5 ng μl^−1^of polybrene for 30 min at 37 °C. We then added the pseudotype/antibody mixture to cells for 3 h, and then replaced media with DMEM supplemented with 10% (v/v) FBS. We measured entry levels by flow cytometry 48 h post transduction using a LSR-II analyzer (BD biosciences). Relative entry of pseudotypes was calculated as follows: Relative entry (%) = (%GFP-positive cells in the presence of antibody/%GFP-positive cells in the absence of antibody) × 100. Antibody neutralization of pseudotypes was calculated as follows: Neutralization (%) = [1−(%GFP-positive cells in the presence of antibody/%GFP-positive cells in the absence of antibody)] × 100.

### ELISA experiments

We used SA-coated ELISA plates (Thermo Fisher Scientific) and coated wells with antigens (JUNV GP1, JUNV GP1_mut_, MACV GP1, or LUJV GP1, all biotinylated at an N-terminal BirA ligase site) at a concentration of 2 μg ml^−1^ in PBS containing 1% (v/v) bovine serum albumin (BSA). For binding assays, we added IgG at increasing concentrations and incubated wells for 1 h. We detected bound antibody with horseradish peroxidase (HRP)-coupled anti-human (Fab) antibody (Sigma-Aldrich catalog number A0293). For ELISA-based competition assays, we added CR1-07 IgG, CR1-10 IgG, or CR1-28 IgG (which have a His_6_-tag at their C termini) at a fixed concentration during a pre-incubation step of 30 min to obtain a baseline signal of 1.5–2.5 OD 450 nm. We then added GD01 IgG or 17b IgG at increasing concentrations. We detected bound antibody using (HRP)-coupled anti-human (Fab) antibody (Sigma-Aldrich catalog number A0293).

### Cell surface GPC expression and antibody staining

We used Lipofectamine 3000 (Invitrogen) to transfect HEK293T cells with a previously described JUNV GPC-expressor plasmid, which we also used for pseudotype production.^10^ We harvested cells 48 h post-transfection in PBS containing 1 mM EDTA using a cell scraper and passed cells through a cell strainer. After a blocking step in PBS with 4% goat serum for 30 min on ice, we incubated cells (transfected or untransfected) with increasing concentrations of CR1-28 IgG, CR1-10 IgG, or 17b IgG in PBS containing 2% (v/v) goat serum for 30 min on ice. After this incubation step, we washed cells twice in PBS containing 2% goat serum. We then incubated cells with antibody with a PE-coupled anti-human goat F(ab’)_2_ fragment (Jackson ImmunoResearch catalog number 109-116-098) for 1 h, washed the cells twice in PBS containing 2% goat serum, passed them through a cell strainer, and detected bound antibody by FACSymphony analyzer (BD Biosciences).

### Surface plasmon resonance-binding assays

We performed binding experiments in duplicate with a Biacore 3000 (GE Healthcare Life Sciences) using SA-coated sensor chips and JUNV GP1 or MACV GP1 biotinylated at an N-terminal BirA ligase site. We captured ~600 response units of biotinylated proteins onto the chip to avoid rebinding events. We carried out experiments in HBS-EP [10 mM HEPES pH 7.5, 150 mM NaCl, 3 mM EDTA, and 0.005% (v/v) P-20]. We passed Fabs over the surface at different concentrations. The concentrations for JUNV GP1-coated surfaces were; CR1-07 Fab 500 nM, 250 nM, 125 nM, 62.5 nM, or 31.25 nM; CR1-09 Fab 400 nM, 200 nM, 100 nM, 50 nM, or 25 nM; CR1-10 Fab 25 nM, 12.5 nM, 6.25 nM, 3.125 nM, or 1.5625 nM; CR1-28 Fab 100 nM, 50 nM, 25 nM, 12.5 nM, or 6.25 nM. The concentrations for MACV GP1-coated surfaces were; CR1-07 Fab 500 nM, 250 nM, 125 nM, 62.5 nM, or 31.25 nM. We analyzed GP1–Fab interactions using multi-cycle kinetic analysis with 2 min association and 5 min dissociation phases with a flow rate of 50 μl min^−1^. We regenerated the surface between each cycle with two 5 μl injections of 35 mM NaOH, 1.3 M NaCl at 100 μl min^−1^, and 2 min stabilization after regeneration. For analysis, we subtracted injections over blank surfaces from the data, and fit the data using a 1:1 Langmuir binding model in the BiaEvaluation software (GE Healthcare Life Sciences).

### Plaque reduction neutralization tests

We seeded Vero cells at 100,000 per well in 12-well plates the day before the experiment. We mixed 80 plaque-forming units of the respective infectious viruses with serially diluted antibodies in minimal essential medium (MEM) (Life Technologies) supplemented with 2% (v/v) FBS and 1% penicillin-streptomycin (P/S) (Life Technologies). After incubation for 1 h at 37 °C, the mixture was inoculated onto Vero cells and incubated for 1 h at 37 °C. Temin’s 2× MEM containing 4% (v/v) FBS and 2% (v/v) P/S was mixed with equal volume of 1.2% gum tragacanth (Sigma-Aldrich) and used as an overlay medium. We incubated plates for 7 days and fixed and stained plates with 1% (v/v) crystal violet in 10% (v/v) formalin. Percent plaque number was calculated as follows: Plaque number (%) = (plaque number in the presence of antibody/plaque number in the absence of antibody) × 100.

### Data collection and structure determination

A CR1-10 Fab/JUNV GP1/CR1-28 Fab complex co-eluted on a S200 size-exclusion column. We concentrated the complex in buffer containing 25 mM Tris, pH 7.5, and 150 mM NaCl (GFB) to a final protein concentration of 35 OD ml^−1^. Crystals grew in 1.9 M ammonium sulfate pH 7.7 and were flash frozen in well solution containing 15% (v/v) glycerol. The complex crystallized in the *P*2_1_ space group with 4 copies in the ASU. We collected X-ray diffraction data at a wavelength of 0.979 and temperature of 100 K on NE-CAT APS beamline 24-ID-E at the advanced photon source (APS, Argonne National Laboratory). We processed data using XDS and XSCALE,^55^ and determined the structure of the complex by molecular replacement with PHASER^56^ using JUNV GP1 (PBD: 5EN2)^14^ as a search model and BALBES to identify the Fab fragments.^28^ We concentrated the CR1-07 Fab in GFB to a concentration of 25 mg ml^−1^ and obtained crystals in mother liquor containing 19% (v/v) polyethylene glycol monomethyl ether 550, MES pH 6.5 and 10 mM zinc sulfate heptahydrate. The Fab crystallized in the *C*2 space group with one copy in the ASU. We collected X-ray diffraction data at a wavelength of 0.979 and temperature of 100 K on NE-CAT APS beamline 24-ID-C (APS). We processed data using XDS and XSCALE^55^ and determined the structure by molecular replacement with BALBES.^28^ We concentrated the MACV GP1/CR1-07 Fab complex to a final concentration of 35 OD ml^−1^ in GFB. Crystals grew in 1.8 M ammonium sulfate and 100 mM sodium citrate pH 5.5. The complex crystallized in the *P*4_2_2_1_2 space group. We collected X-ray diffraction data at a wavelength of 0.979 and temperature of 100 K at NE-CAT APS beamline 24-ID-E. We processed data using XDS and XSCALE,^55^ and determined the structure of the complex by molecular replacement with PHASER^56^ with MACV GP1 (PBD: 2WFO)^18^ and the unliganded CR1-07 Fab as a search models. For all structures, we performed iterative model building with COOT^57^ and refinement with PHENIX^58^ and/or BUSTER.^59^ For both the CR1-10 Fab/JUNV GP1/CR1-28 Fab and the MACV GP1/CR1-07 Fab complex, four copies of the complex were identified per ASU, allowing for electron density modification for phase improvement, which we performed using Parrot in the CCP4 software suite.^60^ We decided on resolution cut off by considering completeness of the data, statistically significant CC_1/2_^61^ and map quality and interpretability. Sample stereo images of electron density maps and snapshots of the maps at the interfaces are included in Supplementary Figure 4, 6 and 9. Data collection and refinement statistics are listed in Supplementary Table 3. We used SBGrid-supported applications to complete our structural studies,^62^ validated structures using MolProbity,^63^ and made figures with the PyMol Molecular Graphics System, Schrödinger, LLC.

### Sample size determination

ELISAs in Figs. 1 and 2 and in Supplementary Figure 3 were done in duplicate twice (*n* = 2) with representative data shown. Neutralization and entry assays shown in Figs. 1 and 2 were done twice in duplicate (*n* = 2), and data are shown as averaged for the two experiments. For the cell-binding experiment done in Supplementary Figure 5, the experiment was done in duplicate twice (*n* = 2) and representative data from one experiment are shown. For the studies done with live infectious viruses in Fig. 2, the experiment was done once in duplicate (*n* = 1). We chose these sample sizes based on similar studies published in the literature and because experiments done in this fashion allow for appropriate qualitative assessments of antibody activity, in particular when comparing different antibodies against each other or different viruses with appropriate controls.

### Data availability

All relevant data are available from the authors upon request. The accession numbers for the coordinates and structure factors reported for the CR1-10 Fab/JUNV GP1/CR1-28 Fab complex, the MACV GP1/CR1-07 Fab complex, and the CR1-07 unliganded Fab are Protein Data Bank (PDB) 5W1K, 5W1M, and 5W1G, respectively. The GenBank accession numbers for the V_H_ and Vκ antibody gene segment sequences respectively are MH151333 and MH151334 for CR1-07, MH151335 and MH151336 for CR1-10, and MH151337 and MH151338 for CR1-28.

## Electronic supplementary material

Supplementary Information
