# Supplementary material for: Vaccine-elicited receptor-binding site antibodies neutralize two New World hemorrhagic fever arenaviruses
Source: Nat Commun. 2018 May 14;9:1884. doi: 10.1038/s41467-018-04271-z (PMC5951886; doi:10.1038/s41467-018-04271-z)
Supplement: Supplementary file 1 — Supplementary Information [file 41467_2018_4271_MOESM1_ESM.pdf]

Supplementary Information

Vaccine-elicited receptor-binding site antibodies neutralize two New World hemorrhagic fever arenaviruses

Lars E. Clark[1#], Selma Mahmutovic[2#], Donald D. Raymond[2], Takaaki Koma[3], John T. Manning[3], Taleen Dilanyan[1], Sundaresh Shankar[1], Silvana C. Levis[4], Ana M. Briggiler[4], Delia A. Enria[4], Kai W. Wucherpfennig[1,5,6], Slobodan Paessler[3], Jonathan Abraham[1,2,7]*

[1]Department of Microbiology and Immunobiology, Harvard Medical School, Boston, MA 02115, USA; [2]Laboratory of Molecular Medicine, Boston Children's Hospital, Harvard Medical School, Boston, MA 02115, USA; [3]Department of Pathology, University of Texas Medical Branch at Galveston, Galveston, TX 77555, USA; [4]Instituto Nacional de Enfermedades Virales Humanas "Dr. Julio I Maiztegui", Monteagudo 251 Pergamino, Buenos Aires 2700, Argentina; [5]Department of Cancer Immunology and Virology, Dana-Farber Cancer Institute, Boston, MA 02115, USA; [6]Program in Immunology, Harvard Medical School, Boston, MA 02115, USA; [7]Department of Medicine, Division of Infectious Diseases, Brigham and Women's Hospital, Boston, MA 02115, USA.

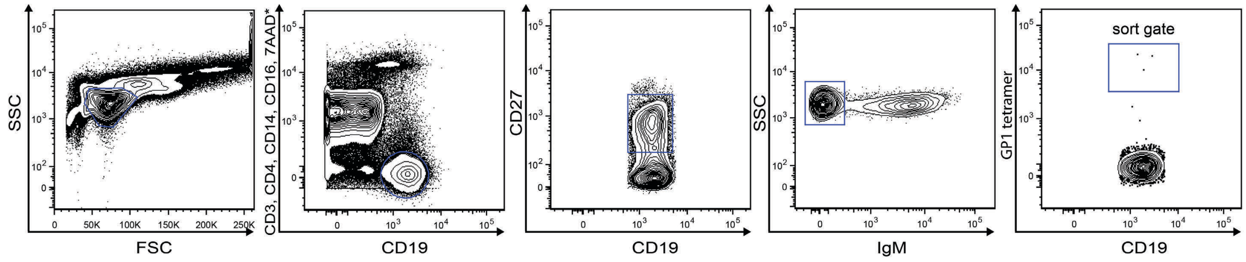

Supplementary Figure 1. Gating scheme for single B cell sorting. Gating scheme for isolation of antigen-specific memory B cells ($CD19^+$, $CD27^+$, IgM^-, JUNV $GP1^+$). Asterisk indicates the exclusion channel; in Sort 2 (Fig. 1c), the exclusion channel also included cells that stained positive for JUNV $GP1_{mut}$-PerCP.

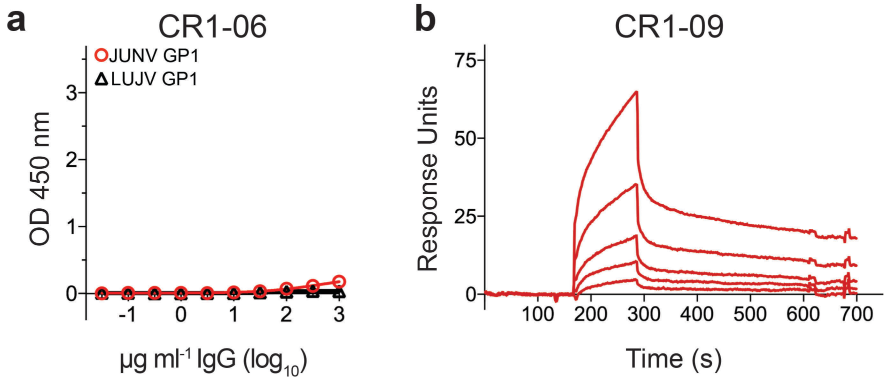

Supplementary Figure 2. Analysis of CR1-06 and CR1-09 binding to JUNV GP1. (a) ELISA of CR1-06 IgG binding to immobilized JUNV GP1. LUJV GP1 is a control. Error bars, which indicate standard deviation, are smaller than symbols. (b) Sensorgrams for binding of CR1-09 Fab to immobilized JUNV GP1 as measured by surface plasmon resonance. CR1-09 Fab was passed over the surface at concentrations of 400 nM, 200 nM, 100 nM, 50 nM, and 25 nM. The recorded sensorgrams (one of duplicates) are shown. The fast off rate prevented accurate fitting of the data to estimate binding kinetics.

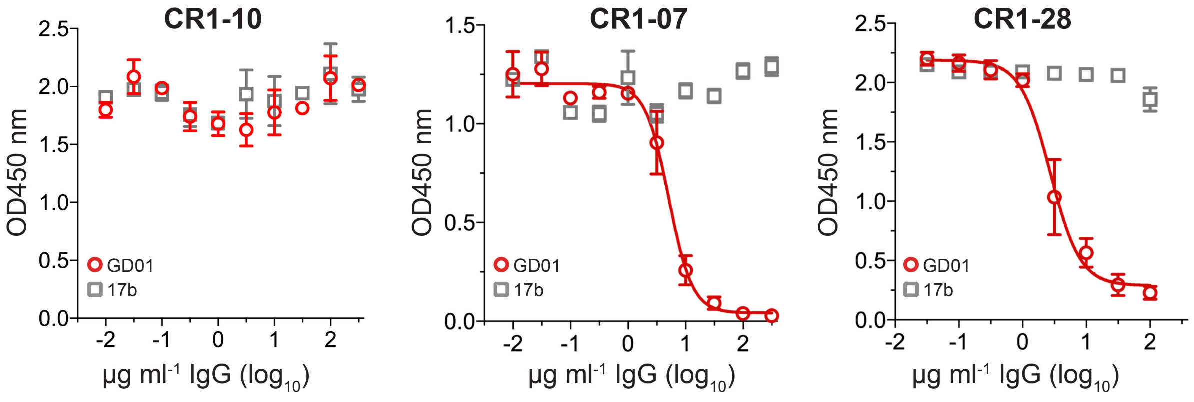

Supplementary Figure 3. CR1-07 and CR1-28 compete with GD01 for binding to JUNV GP1. Competitor IgG (GD01 or 17b) was added at increasing concentrations to plates coated with JUNV GP1. After an incubation step, CR1-10 IgG (left panel), CR1-07 IgG (middle panel), or CR1-28 IgG (right panel) were added at fixed concentrations. After several wash steps, CR1-07, CR1-

10, or CR1-28 IgG that bound to the plate was detected using an anti-human IgG secondary antibody. The experiment was performed twice in duplicate and representative data are shown. Error bars indicate standard deviation (S.D.).

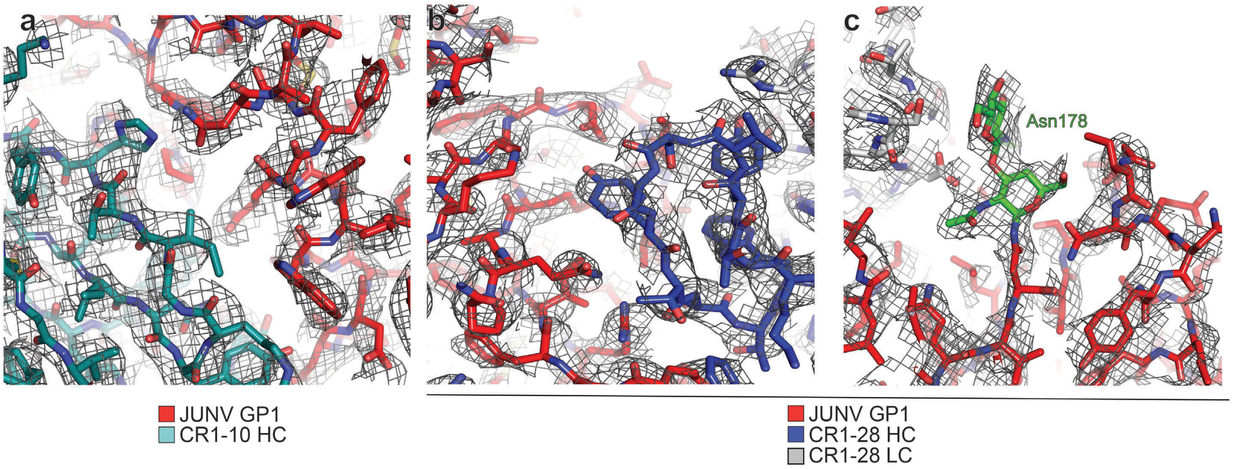

Supplementary Figure 4. Electron density for the CR1-10 Fab/JUNV GP1/CR1-28 Fab complex. (a) Part of the CR1-10 Fab/JUNV GP1 interface is shown as sticks with the refined $2F_o$-F_c electron density at 1 σ. (b) Part of the CR1-28 Fab/JUNV GP1 interface is shown as sticks with the refined $2F_o$-F_c electron density at 1 σ. (c) $2F_o$-F_c electron density at 1 σ for a N-linked glycan (shown as green sticks) attached at JUNV GP1 Asn178.

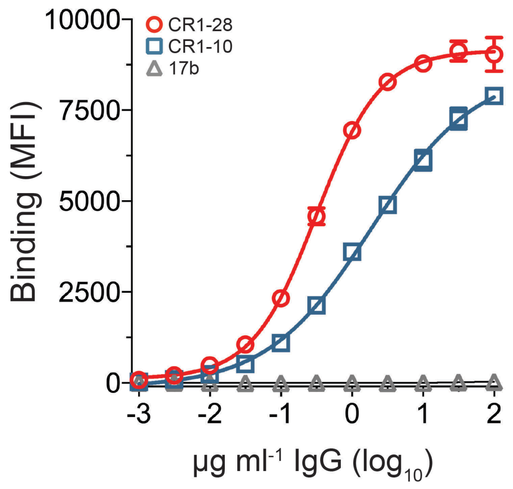

Supplemental Figure 5. CR1-10 binds cell-surface expressed JUNV GPC trimer. We transfected HEK293T cells with JUNV GPC and measured binding of CR1-28 IgG, CR1-10 IgG, or 17b IgG (control) by FACS 48 hr after transfection. Mean fluorescence intensity (MFI) values for antibody-binding to transfected cells were corrected by subtraction of MFI obtained at the same antibody concentration with control, untransfected HEK293T cells. The EC_{50} for CR1-28 and CR1-10 binding to cells transfected with JUNV GPC was measured as 0.32 μg ml^{-1} and 1.9 μg ml^{-1}, respectively. Error bars indicate S.D. For some data points, error bars are smaller than symbols. The experiment was performed twice in duplicate and representative data are shown.

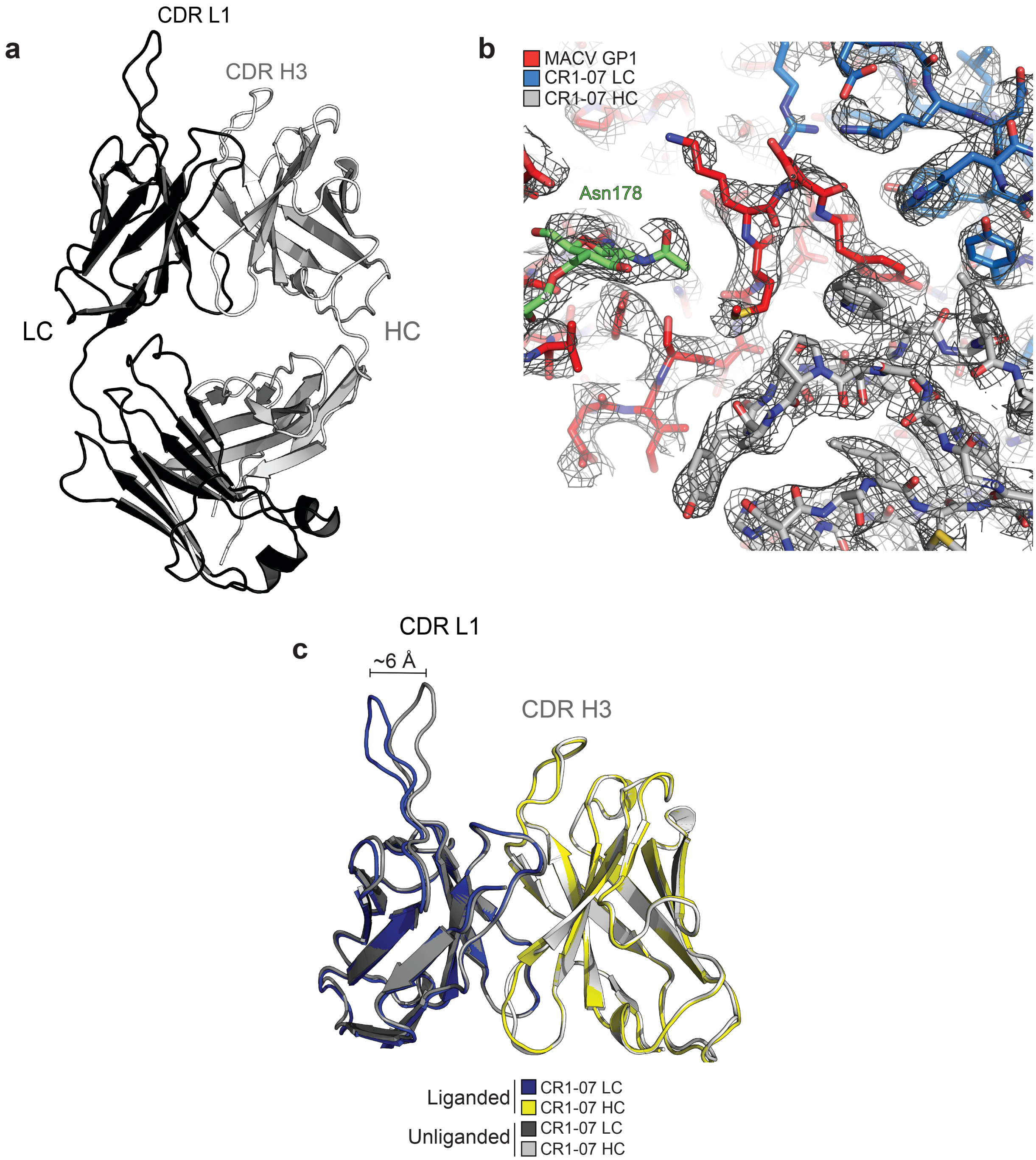

Supplementary Figure 6. CR1-07 Fab structure and electron density for MACV GP1/CR1-07 Fab complex. (a) Ribbon diagram of the unliganded CR1-07 Fab (heavy chain in gray, light chain in black). HC: heavy chain; LC: light chain. (b) Part of the MACV GP1/CR1-07 Fab interface is shown as sticks with the refined $2F_o$-F_c electron density at 1 σ. (c) One of the four Fab copies (representative) found in the asymmetric unit of the MACV GP1/CR1-07 Fab complex was

overlaid onto the unliganded CR1-07 Fab. The tip CR1-07 CDR L1 shifts by about 6 Å to accommodate MACV GP1 in the complex.

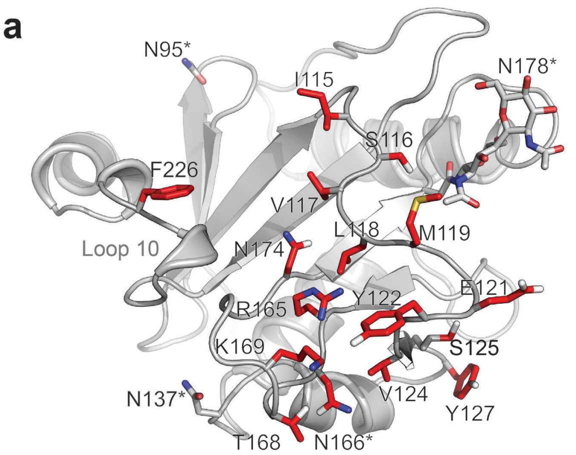

b

Position	MACV	JUNV	TCRV	GTOV	CHPV	SBAV
115	I	I	V	V	M	V
116	S	A	T	H	S	T
117	V	V	V	P	V	L
118	L	L	L	V	L	L
119	M	L	T	L	L	M
121	E	E	S	S	P	E
122	Y	Y	A	S	H	H
124	V	V	V	P	P	R
125	S	I	F	T	R	Q
127	Y	Q	H	L	M	L
165	R	R	R	E	E	Q
166	N	N	N	P	K	K
168	T	T	T	K	T	T
169	K	K	K	T	S	S
174	N	I	L	T	R	R
226	F	-	-	-	-	-

Supplementary Figure 7. CR1-07 contact residues in MACV GP1. (a) Ribbon diagram of MACV GP1 with residues that are contacted by CR1-07 shown as sticks and colored red. Asparagines that are potential sites of N-linked glycan attachment are shown as sticks and indicated with an asterisk. (b) List of CR1-07 contact residues in MACV GP1 and residues at the analogous positions in the GP1s of other New World arenaviruses (using MACV GP1 numbering). Residues highlighted in grey are identical between MACV and the indicated GP1.

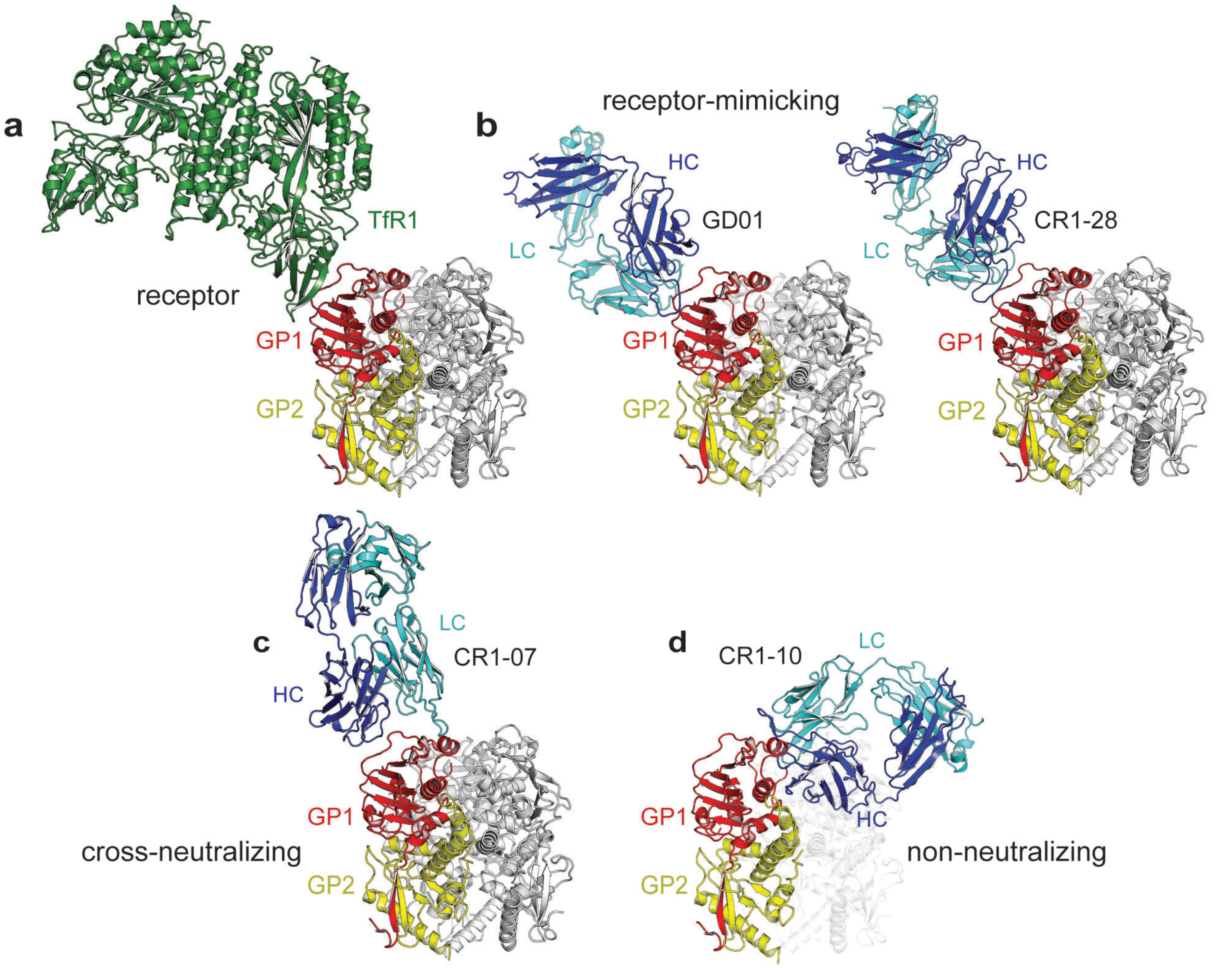

Supplementary Figure 8. Model of neutralizing antibody binding to the pre-fusion arenavirus GPC ectodomain. (a) The MACV GP1/TfR1 complex (PDB: 3KAS)[1] was overlaid onto the structure of the prefusion LASV GPC ectodomain (PDB: 5VK2)[2] by aligning the GP1 proteins, which have a similar fold. One of the three GP1:GP2 protomers is colored for GPC. (b) Left: Structure of the JUNV GP1/GD01 Fab complex (PDB: 5EN2)[3] overlaid onto the prefusion LASV GPC ectodomain structure as in *a.* Right: Structure of the JUNV GP1/CR1-28 Fab subcomplex overlaid onto the LASV GPC ectodomain structure as in *a*. GD01 and CR1-28 are receptor-mimicking antibodies. (c) Structure of the MACV GP1/CR1-07 Fab complex overlaid onto the structure of the LASV GPC as in *a.* CR1-07 is a JUNV/MACV cross-neutralizing antibody. (d) The JUNV GP1/CR1-10 Fab complex was overlaid onto LASV GPC as in *a*, with two of the protomers in trimeric GPC shown as transparent to allow for better visualization of the binding

site. CR1-10 is a non-neutralizing antibody. For all panels, the New World arenavirus GP1 protein used for structural alignment is omitted for clarity.

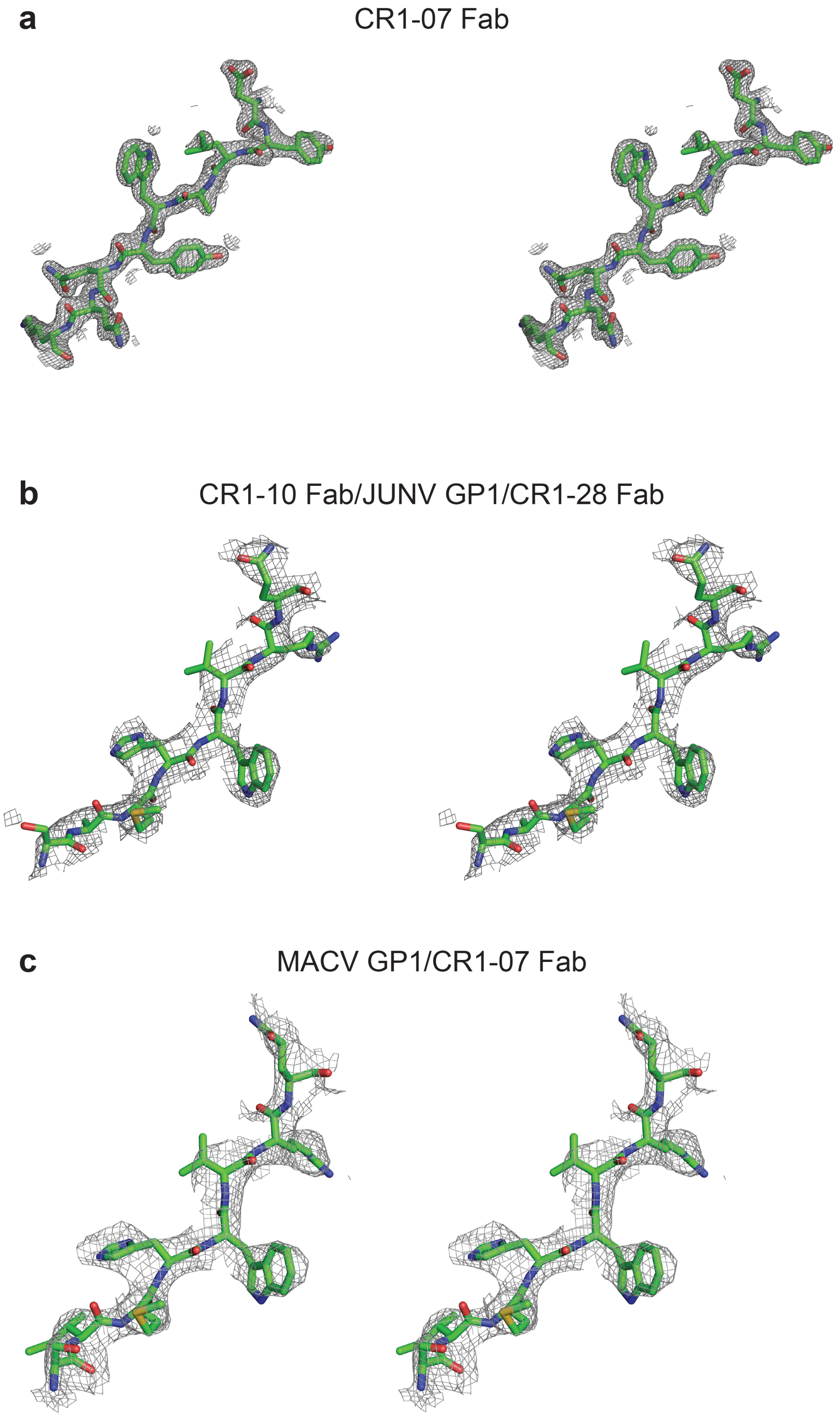

Supplementary Figure 9. Sample electron density for the reported X-ray crystal structures. (a) Sample electron density for the unliganded CR1-07 Fab crystal structure. The volume shown in stereoview is from the $2F_o$-F_c electron density map contoured at 1.5 σ in the region corresponding to light chain residues 39-47. (b) Sample electron density for the CR1-10 Fab/JUNV GP1/CR1-28 Fab crystal structure. The volume shown in stereoview is from the $2F_o$-F_c electron density map contoured at 1.0 σ in the region corresponding to residues 32-39 of the CR1-28 heavy chain (chain T in the asymmetric unit). (c) Sample electron density for the MACV GP1/CR1-07 Fab crystal structure. The volume shown in stereoview is from the $2F_o$-F_c electron density map contoured at 1.0 σ in the region corresponding to CR1-07 Fab heavy chain residues 32-39 (chain F in the asymmetric unit).

Supplementary Table 1. Properties of GP1-directed antibodies.

Antibody	V_H	D	J_H	CDR3	Length	Mutations	% Mutation
CR1-06	3-7	3-10	4/5	AKVGGHSAFYKTGRVGFDS	19	39	13.4%
CR1-07	3-30	3-16/3-9	4	AKDLSPPYSYAWDIFQY	17	39	13.2%
CR1-09	4-4	2-15	4	ARYCSGSACVPFDF	14	27	9.2%
CR1-10	4-31	2-15	4	ARLTSDCSGGNCYQAFDY	18	33	11.1%
CR1-28	3-33	6-13	4	ATDKTYVSGYTSTWYYFNY	19	11	3.7%
*GD01	1-4/1-7	1-1	4	ARRRVYYGSNYIYALDY	17	38	13.0%

	V_k	J_k	CDR3	Length	Mutations	% Mutation
CR1-06	1-12/1D-12	2	QQGNSLPYT	9	39	13.70%
CR1-07	4-1	4	QQYYSSPPT	9	14	6.6%
CR1-09	3-20	1	LQYGTRPRT	9	21	7.3%
CR1-10	3-20	2	QQYGRSGYT	9	9	3.2%
CR1-28	1-5	1	QHRT	4	4	1.5%
*GD01	6-13	5	QQYSSYPLA	9	6	2.1%

*For GD01, murine antibody gene usage patterns are shown.

Supplementary Table 2. Binding rate constants of surface plasmon resonance analysis of antibody Fabs binding to immobilized GP1.

Analyte	Ligand	k_a (1/Ms)	k_d (1/s)	K_D (M)
CR1-07 Fab	JUNV GP1	8.52E+03	7.34E-04	8.62E-08
CR1-10 Fab	JUNV GP1	8.10E+05	3.65E-05	4.50E-11
CR1-28 Fab	JUNV GP1	1.05E+05	5.54E-04	5.30E-09
CR1-07 Fab	MACV GP1	4.13E+04	6.76E-04	1.60E-08

Supplementary Table 3. Data collection and refinement statistics.

	**CR1-07 Fab**	**CR1-10 Fab/JUNV GP1/CR1-28 Fab**	**MACV GP1/CR1-07 Fab**
**Data collection**			
Space group	*C*2	$P2_1$	$P4_22_12$
Cell dimensions			
a, b, c	89.8 137.4 78.7	154.8 132.2 167.4	206.9 206.9 238.23
Resolution (Å)	2.00 (2.07-2.00)[a]	3.99 (4.23-3.99)	3.91 (4.15-3.91)
R_{merge}	0.12 (1.02)	0.72 (1.58)	0.32 (8.38)
$CC_{1/2}$	99.4 (64.6)	79.6 (46.5)	99.5 (21.5)
Avg I/σ_I	9.57 (1.65)	2.34 (1.03)	7.72 (0.52)
Completeness (%)	97.9 (96.7)	99.1 (97.6)	98.8 (96.8)
Average redundancy	4.5 (4.6)	3.4 (3.4)	8.1 (8.1)
**Refinement**			
Resolution (Å)	44.21-2.00	49.35-3.99	49.12-3.91
No. reflections	32,718	57,194	46,614
R_{work}/R_{free}	0.23/0.28	0.24/0.29	0.25/0.26
Number of Atoms			
Protein	3,378	30,971	18,299
Water	223	-	-
R.m.s. deviations			
Bond lengths (Å)	0.005	0.008	0.012
Bond angles (°)	0.646	1.15	1.33
Avg B-factor (Å^2)			
Protein	47.8	69.0	33.5
Water	44.8	-	-

One crystal was used to collect each data set.

[a]Values in parentheses are for highest-resolution shell

Supplementary Table 4. XSCALE[4] output summary.

Subset of intensity data with signal to noise >=-3.0 as function of resolution for **JUNV** GP1 complex

Resolution limit	Reflections Observed	Reflections Unique	Reflections Possible	Completeness of data	R-factor observed	R-factor expected	Compared	I/SIGMA	R-meas	CC(1/2)
11.68	7797	2333	2406	97.00%	13.40%	13.80%	7764	7.53	16.00%	98.8*
8.38	13247	3931	3973	98.90%	16.60%	16.90%	13167	6.55	19.80%	98.2*
6.87	17016	5072	5115	99.20%	45.50%	45.20%	16869	2.91	54.10%	90.4*
5.97	19914	5866	5902	99.40%	79.60%	79.30%	19792	1.84	94.60%	73.2*
5.34	23300	6838	6867	99.60%	83.30%	82.20%	23160	1.78	99.00%	72.4*
4.88	25365	7432	7450	99.80%	73.80%	73.40%	25241	1.97	87.80%	77.0*
4.52	27660	8086	8114	99.70%	81.30%	80.50%	27538	1.85	96.60%	73.9*
4.23	29698	8680	8705	99.70%	102.20%	100.70%	29599	1.57	121.60%	64.4*
3.99	30534	8957	9177	97.60%	158.60%	156.70%	30401	1.03	188.60%	46.5*
Total	194531	57195	57709	99.10%	72.10%	71.40%	193531	2.34	85.70%	79.6*

Subset of intensity data with signal to noise >=-3.0 as function of resolution for **MACV** GP1 complex

Resolution limit	Reflections Observed	Reflections Unique	Reflections Possible	Completeness of data	R-factor observed	R-factor expected	Compared	I/SIGMA	R-meas	CC(1/2)
11.45	14549	2013	2093	96.20%	2.90%	3.60%	14523	46.89	3.10%	99.9*
8.21	24884	3299	3341	98.70%	4.70%	5.00%	24836	33.4	5.00%	99.9*
6.73	33344	4196	4237	99.00%	17.20%	17.30%	33296	12.92	18.40%	99.3*
5.84	39740	4909	4943	99.30%	42.70%	42.80%	39700	6.31	45.60%	95.7*
5.24	44300	5425	5464	99.30%	65.20%	65.30%	44265	4.42	69.60%	92.2*
4.78	50412	6149	6173	99.60%	87.10%	87.00%	50371	3.49	92.90%	88.1*
4.43	53993	6554	6581	99.60%	153.20%	152.80%	53962	2.15	163.30%	75.1*
4.15	57007	6911	6937	99.60%	262.50%	261.40%	56980	1.25	279.70%	49.5*
3.91	59863	7376	7621	96.80%	838.00%	834.60%	59833	0.52	894.00%	21.5*
total	378092	46832	47390	98.80%	32.90%	33.10%	377766	7.72	35.10%	99.5*

Definitions

Compared	Number of reflections used for calculating R-factor
I/SIGMA	Mean of intensity/Sigma(I) of unique reflections (after merging symmetry-related observations)
Sigma(I)	Standard deviation of reflection intensity I estimated from sample statistics

R-meas	Redundancy independent R-factor (intensities)
CC(1/2)	Percentage of correlation between intensities from random half-datasets. Correlation significant at the 0.1% level is marked by an asterisk.
Anomal Corr	Percentage of correlation between random half-sets of anomalous intensity differences. Correlation significant at the 0.1% level is marked.
SigAno	Mean anomalous difference in units of its estimated standard deviation (|F(+)-F(-)|/Sigma). F(+), F(-) are structure factor estimates obtained from the merged intensity observations in each parity class.
Nano	Number of unique reflections used to calculate Anomal_Corr & SigAno. At least two observations for each (+ and -) parity are required.

Supplementary References

1. Abraham, J. *et al.* Structural basis for receptor recognition by New World hemorrhagic fever arenaviruses. *Nature Structural & Molecular Biology* **17,** 438–444 (2010).
2. Hastie, K. M. *et al.* Structural basis for antibody-mediated neutralization of Lassa virus. *Science* **356,** 923–928 (2017).
3. Mahmutovic, S. *et al.* Molecular Basis for antibody-mediated neutralization of New World hemorrhagic fever mammarenaviruses. *Cell Host & Microbe* **18,** 705–713 (2015).
4. Kabsch, W. XDS. *Acta Crystallographica. Section D, Biological Crystallography* **66,** 125–132 (2010).
